# [Corrigendum] Investigating the efficacy of tissue factor pathway inhibitor‑2 as a promising prognostic marker for ovarian cancer

**DOI:** 10.3892/ol.2025.14978

**Published:** 2025-03-17

**Authors:** Tomoka Maehana, Ryuji Kawaguchi, Kyohei Nishikawa, Naoki Kawahara, Yuki Yamada, Fuminori Kimura

Oncol Lett 28: 302, 2024; DOI: 10.3892/ol.2024.14435

Following the publication of this article, the authors have realized that there were a few errors contained therein that they neglected to correct before the paper was published. These are listed as follows:

i) First, in the Abstract, the sentences commencing on line 17, some factually incorrect information was provided. The remainder of the Abstract should have read as follows (the changed text is highlighted in **bold**): “In univariate analysis, OS was **inferior** in patients with non-OCCC and OCCC exhibiting TFPI2 levels ≥201 pg/ml (P<0.001) and ≥255 pg/ml (P=0.036), respectively. Similarly, progression-free survival (PFS) was significantly **inferior** in patients with non-OCCC and OCCC with TFPI2 levels ≥201 pg/ml and ≥255 pg/ml (both P<0.001), respectively. Multivariate analysis further confirmed that OS was significantly **inferior** in patients with non-OCCC who had TFPI2 levels ≥201 pg/ml (P=0.021), whereas PFS was **inferior** in patients with OCCC who had TFPI2 levels ≥255 pg/ml (P=0.020). These findings indicate that TFPI2 may serve as a potential prognostic biomarker for ovarian carcinoma.

ii) Secondly, the following revision of the text was necessary for the Discussion, p. 5, right-hand column, final paragraph, the second sentence: “TFPI2 levels ≥201 pg/ml for predicting OS for non-OCCC and ≥255 pg/ml for predicting **OS for OCCC** were the cut-off values.”

The authors sincerely apologize for the errors that were present in the text for the Abstract and Discussion, thank the Editor of *Oncology Letters* for allowing them the opportunity to publish this Corrigendum, and regret any inconvenience that these mistakes may have caused.

